# Delayed Duodenal Ulcer Perforation Following Esophageal Endoscopic Submucosal Dissection Complicated by Perforation: A Case Report

**DOI:** 10.1002/deo2.70269

**Published:** 2026-01-21

**Authors:** Shinya Nakatani, Sayaka Mizuno, Takahiro Fuji, Yoshinao Onishi, Kazuya Inoki, Masayuki Tojo, Kunihiko Wakamura, Atsushi Katagiri, Takeshi Aoki, Hitoshi Yoshida

**Affiliations:** ^1^ Department of Medicine Division of Gastroenterology, Showa Medical University School of Medicine Tokyo Japan; ^2^ Endoscopy Center Showa Medical University Hospital Tokyo Japan; ^3^ Department of Surgery Division of Gastroenterological and General Surgery, Showa Medical University School of Medicine Tokyo Japan

**Keywords:** duodenal ulcer, esophageal endoscopic submucosal disection, perforation, proton pump inhibitors, stress ulcer

## Abstract

Endoscopic submucosal dissection (ESD) is an established treatment of superficial esophageal neoplasms. Common complications include bleeding, perforation, and stricture. However, delayed gastrointestinal perforation distant from the ESD site is exceptionally rare. We report the case of a woman in her 70s with a history of nonsteroidal anti‐inflammatory drug (NSAID)‐associated duodenal ulcer and *Helicobacter pylori* infection who underwent ESD for superficial esophageal squamous cell carcinoma. After eradication therapy and 2 months of proton pump inhibitor (PPI) use, both treatments were discontinued. Preoperative endoscopy confirmed a scarred duodenal ulcer. Intraoperative esophageal perforation occurred during ESD and was closed with clips. Postoperative computed tomography (CT) showed mediastinal emphysema without intra‐abdominal free air. The patient was treated in high care with fasting and antibiotics, but without PPI therapy. Six days postoperatively, the patient developed acute abdominal pain. CT revealed free air near the duodenal bulb, and emergency endoscopy identified a 10‐mm perforated duodenal ulcer at the scarred site. Endoscopic closure was unfeasible, and laparoscopic omental patch repair was performed. PPI therapy was resumed postoperatively, and the patient recovered uneventfully. This case suggests that stress‐related mucosal disease may have contributed to duodenal perforation. Background risks included ulcer history and scarring, whereas alleviating factors included no NSAID/steroid exposure, eradicated *H. pylori*, and absence of infection at the esophageal perforation. Guidelines do not endorse routine PPI use after ESD, and consensus following iatrogenic perforation is lacking. This case suggests that prophylactic PPI therapy may be considered in patients with risk factors such as recent peptic ulcer disease or intraoperative perforation.

## 1 Introduction

Endoscopic submucosal dissection (ESD) is the standard treatment for early‐stage esophageal squamous cell carcinoma (SCC), allowing en bloc resection and precise histological assessment. Although safe for experienced surgeons, ESD is associated with procedural risks, particularly intraoperative perforation. Perforation during esophageal ESD occurs in approximately 1.4%–4.6% of cases, and can typically be managed conservatively if promptly detected [1, 2].

The current Japan Esophageal Society guidelines do not recommend administration of proton pump inhibitors (PPIs) following esophageal ESD [3]. Unlike gastric ESD, in which acid suppression is essential for ulcer healing and bleeding prevention, esophageal ESD ulcers generally heal spontaneously. Several randomized observational studies have shown no significant benefit of PPI therapy for ulcer healing, with no prevention of delayed bleeding or esophageal perforation [4, 5].

Furthermore, for iatrogenic esophageal perforation, no clear recommendations involving PPIs exist. The European Society of Gastrointestinal Endoscopy (ESGE) guidelines [6] explicitly recommend PPI therapy in such cases, whereas neither the Japanese nor the American guidelines provide guidance, highlighting a discrepancy in practice standards.

We present a rare case of delayed duodenal perforation following ESD‐induced esophageal perforation, suggesting the potential role of stress‐related mucosal disease (SRMD) and emphasizing the need for individualized acid suppression strategies after esophageal ESD with complications.

## 2 Case Report

**FIGURE 1:**
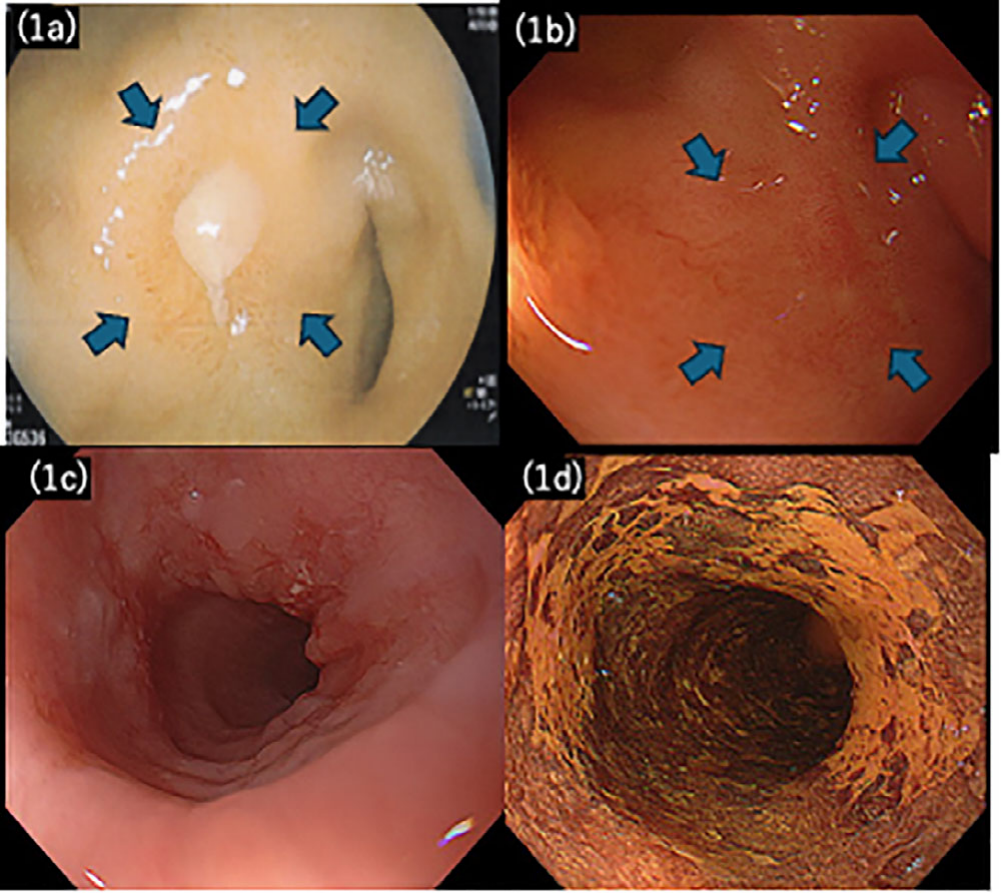
Initial and follow‐up endoscopic findings. (a) Initial esophagogastroduodenoscopy (EGD) at the referring hospital showing an active duodenal bulb ulcer. (b) Findings of the follow‐up endoscopy at our hospital after *Helicobacter pylori* eradication and 2 months of PPI therapy, confirming ulcer healing with scarring. (c) White‐light endoscopy showing a 40‐mm superficial depressed lesion (0‐IIc) in the mid‐thoracic esophagus. (d) Chromoendoscopy with iodine staining demonstrating a clear iodine‐voiding area with distinct margins.

A woman in her 70s, with a history of hypertension, presented with epigastric pain. She reported a daily alcohol intake of approximately 300 mL of distilled spirits. She regularly consumed nonsteroidal anti‐inflammatory drugs (NSAIDs) for right shoulder pain. Emergent esophagogastroduodenoscopy (EGD) at a previous hospital revealed an active duodenal bulb ulcer (Figure 1a), with positivity for *Helicobacter pylori* testing. NSAIDs were discontinued, and PPI therapy was administered for 2 months. PPIs were discontinued after ulcer healing, and *H. pylori* eradication was confirmed using a urea breath test.

During endoscopic evaluation, a 40‐mm superficial depressed lesion was identified 25 cm from the incisors in the mid‐thoracic esophagus. Histopathological examination of a biopsy specimen revealed SCC. After 5 months, the patient was referred to our hospital for further evaluation of the duodenal ulcer and definitive cancer treatment.

Upper gastrointestinal endoscopy revealed a 40‐mm 0‐IIc mid‐thoracic esophagus lesion, involving approximately 3/4 of the luminal circumference, with a pink color sign (Figure 1c,d). Narrowband imaging and iodine staining supported the superficial esophageal SCC diagnosis. The duodenal bulb ulcer was scarred, indicating healing (Figure 1b).

The esophageal lesion was deemed suitable for ESD, and the patient was admitted for treatment 1 month after the initial endoscopic evaluation.

**FIGURE 2:**
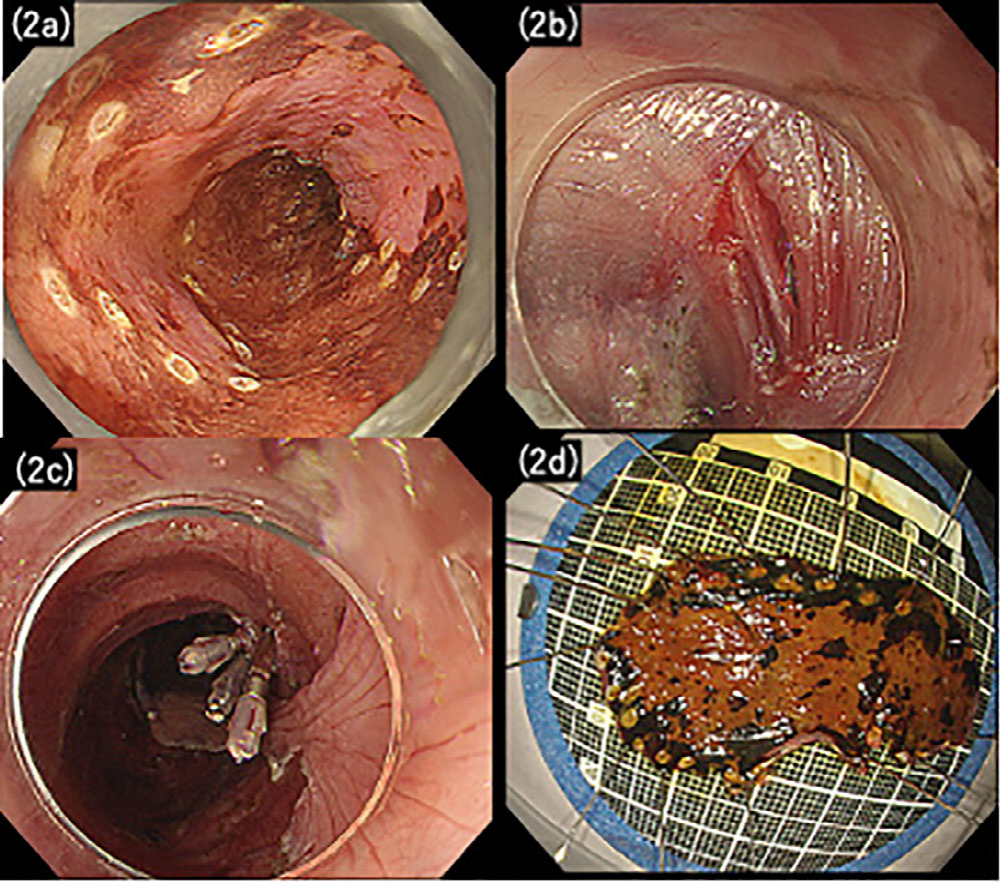
Endoscopic and specimen findings during esophageal endoscopic submucosal dissection (ESD). (a) The endoscopic view during ESD showing marking dots placed around the lesion. (b) The muscular layer incision and perforation identified on the right lateral wall of the esophageal lesion. (c) Complete closure of the perforation with endoscopic clips. (d) The resected esophageal lesion specimen.

On day 2 of hospitalization, ESD was performed under conscious sedation using midazolam, pethidine, and dexmedetomidine, with an IT Knife nano (Olympus Medical Systems, Tokyo, Japan). During submucosal dissection, injury to the muscularis propria caused a visible perforation. The defect was immediately closed with three endoscopic clips (Figure 2a–d). Triamcinolone and systemic corticosteroids were withheld to avoid infection and delayed healing. The total procedural time was 110 min.

**FIGURE 3:**
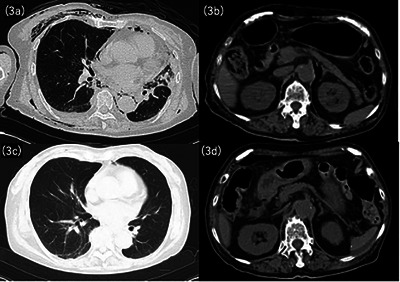
Computed tomography (CT) findings immediately after intraoperative perforation and at the time of postoperative duodenal ulcer perforation. (a) Chest CT immediately after intraoperative perforation, showing subcutaneous and mediastinal emphysema. (b) Abdominal CT immediately after intraoperative perforation, showing retroperitoneal emphysema due to endoscopic submucosal dissection (ESD) perforation without intra‐abdominal free air. (c) Chest CT 6 days postoperatively, demonstrating resolution of mediastinal and subcutaneous emphysema. (d) Abdominal CT 6 days postoperatively, showing the disappearance of retroperitoneal emphysema, but revealing intra‐abdominal free air and fluid collection at the posterior aspect of the duodenal bulb.

Post‐procedural computed tomography (CT) revealed subcutaneous and mediastinal emphysema, without signs of widespread leakage (Figure 3a,b). The Sequential Organ Failure Assessment (SOFA) score remained 0 throughout the perioperative period. Although the patient was hemodynamically stable, she underwent 2 days of high care unit observation in case of delayed deterioration after intraoperative perforation. Postoperatively, she was kept fasting and received intravenous meropenem.

Although inflammatory marker levels transiently increased, C‐reactive protein (CRP) concentration rose from 0.06 mg/dL on admission to 12.8 mg/dL on day 3, then declined to 4.44 mg/dL on day 5, accompanied by gradual improvement in chest pain. However, 6 days postoperatively, while still on bowel rest, the patient developed sudden epigastric pain, coinciding with a CRP level increase to 15.34 mg/dL.

**FIGURE 4:**
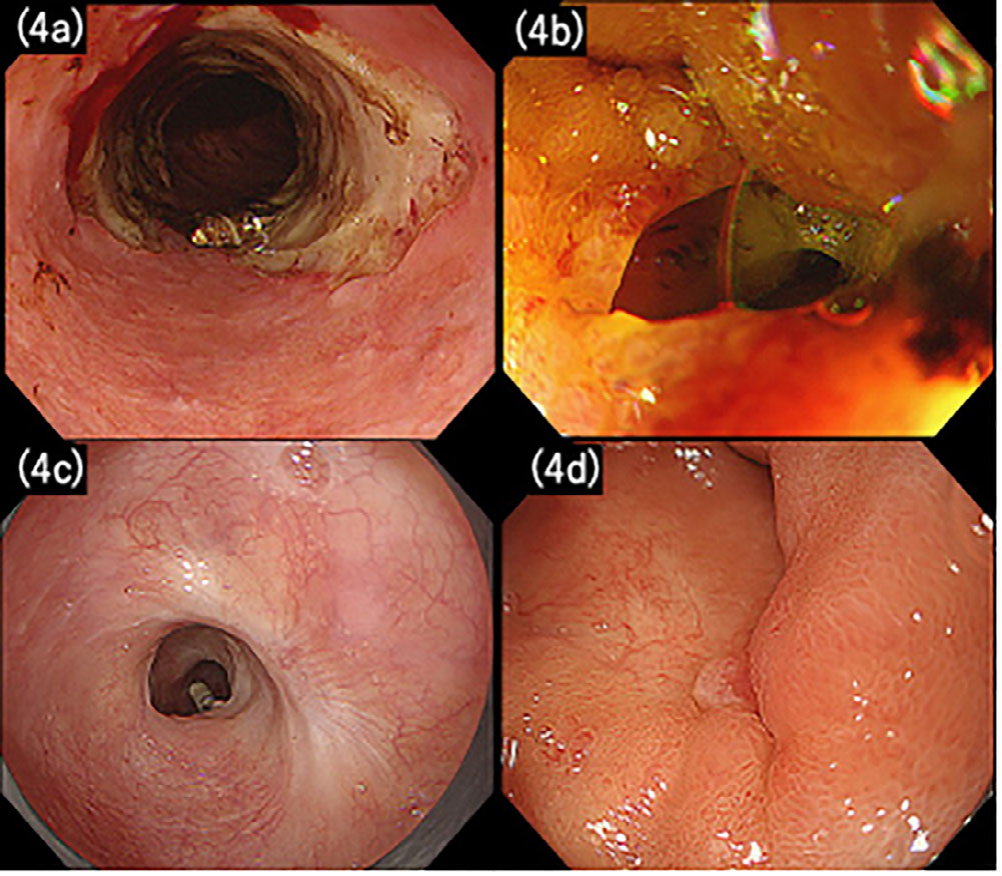
Endoscopic findings 6 days postoperatively and at 3‐month follow‐up. (a) Upper gastrointestinal endoscopy 6 days postoperatively, showing the post‐endoscopic submucosal dissection (post‐ESD) esophageal ulcer with clips in place and no evidence of perforation. (b) Upper gastrointestinal endoscopy 6 days postoperatively, showing a 10‐mm perforation at the duodenal bulb. (c) Follow‐up endoscopy at 2 months, demonstrating scarring of the esophageal ulcer without evidence of recurrence. (d) Follow‐up endoscopy at 2 months showing the duodenal bulb with scarring and mild stenosis, but no evidence of obstruction.

Abdominal CT revealed free intraperitoneal air and fluid collection posterior to the duodenum (Figure 3c,d). EGD was performed to ascertain whether the perforation was related to the previous ESD site and to attempt endoscopic closure if feasible. No leakage was observed in the esophageal ulcers (Figure 4a). However, a 10‐mm perforation was identified at the scarred duodenal bulb, with rigid surrounding mucosa, and endoscopic closure was deemed unfeasible (Figure 4b and Video S1).

The same day, laparoscopic omental patch repair was successfully performed. ESD pathology confirmed pT1a‐LPM SCC with negative horizontal and vertical margins (R0 resection).

The patient resumed oral intake on day 10 and was discharged on day 18, when the CRP concentration had decreased to 2.2 mg/dL, consistent with clinical recovery.

Two months postoperatively, a follow‐up EGD revealed scar formation in both the esophagus and duodenum (Figure 4c,d), with mild stenosis but adequate scope passage. Subsequent annual surveillance endoscopies demonstrated no recurrence, and the patient remained in good condition for 3 years.

## 3 Discussion

This case illustrates a rare sequence of intraoperative esophageal perforation during ESD followed by delayed duodenal ulcer perforation. Preoperative endoscopy confirmed a scarred duodenal ulcer with no active inflammation and no clinical or endoscopic findings suggesting malignancy, duodenitis, Crohn's disease, vasculitis, or ischemia. There was no NSAID or corticosteroid exposure, and *H. pylori* had been eradicated. CT immediately after ESD demonstrated mediastinal and subcutaneous emphysema without intra‐abdominal free air, excluding peritoneal contamination at that time. The overall clinical picture may suggest SRMD as one possible cause of delayed duodenal perforation in this case.

SRMD develops owing to mucosal ischemia and acid hypersecretion under severe systemic stress, including prolonged fasting, infection, and postoperative inflammation. It is most commonly observed in critically ill patients; however, patients with underlying gastrointestinal risk factors, particularly a history of peptic ulcer disease within the preceding year or residual scarring, may be susceptible [7, 8, 9]. SRMD can lead to clinically significant upper gastrointestinal bleeding and, in rare cases, perforation. Therefore, stress ulcer prophylaxis is recommended in high‐risk hospitalized patients [7, 8, 9].

PPI administration after esophageal ESD is not recommended in Japanese guidelines because post‐ESD esophageal ulcers typically heal spontaneously, and clinical studies demonstrated no prevention of delayed bleeding or perforation [3, 4, 5]. In this case, PPI therapy was not initiated immediately after intraoperative perforation because complete endoscopic closure was achieved. The perforation was mid‐esophageal, where gastric acid exposure is relatively limited, and no intra‐abdominal contamination occurred. The patient remained hemodynamically stable with low SOFA scores (0 points), suggesting a low systemic stress burden at that time. Furthermore, to our knowledge, no prior reports have documented delayed perforation of another gastrointestinal segment after esophageal ESD, and SRMD‐related duodenal perforation was not anticipated in this context. Management priorities, therefore, focused on infection control and bowel rest.

This case suggests that in the context of severe procedural stress, prolonged fasting, or post‐procedural monitoring, PPI therapy may be warranted for the protection of gastrointestinal segments other than the esophagus. Current guidelines and reviews addressing ESD complications focus on esophageal healing and perforation management. However, they do not discuss stress ulcer prophylaxis in the context of systemic stress after ESD‐related perforation [10]. Thus, duodenal stress ulcer perforation represents a potential complication not addressed by existing evidence or recommendations.

This case has limitations. First, this is a single case report, and it remains unclear whether prophylactic PPI therapy would have prevented the duodenal perforation. Second, although stress‐related mucosal injury was suspected, the possibility of incidental perforation at a previously scarred site cannot be excluded. Accumulation of additional similar cases is needed to clarify these points.

In conclusion, this case underscores the importance of individualized risk assessments after esophageal ESD. Although routine PPI use is not recommended, selective prophylaxis may be considered in patients with risks, such as recent peptic ulcer disease, intraoperative perforation, prolonged fasting, or intensive care management.

## Author Contributions

**Shinya Nakatani**: conceptualization, writing – original draft, and supervision. **Sayaka Mizuno**: data curation. **Takahiro Fuji**: data curation. **Yoshinao Onishi**: data curation. **Kazuya Inoki**: writing – review and editing. **Masayuki Tojo**: writing – review and editing. **Kunihiko Wakamura**: visualization and writing – review and editing. **Takeshi Aoki**: visualization and writing – review and editing. **Atsushi Katagiri**: visualization and writing – review and editing. **Hitoshi Yoshida**: supervision and writing – review and editing.

## Conflicts of Interest

The authors declare no conflicts of interest.

## Funding

The authors have nothing to report

## Ethics Statement

All procedures were performed in accordance with the ethical standards of the 1964 Declaration of Helsinki and its later amendments.

## Consent

Written informed consent was obtained from the patient for the publication of this case report and accompanying images. This manuscript was prepared in accordance with CARE (CAseREport) guidelines.

## Clinical Trial Registration

N/A

## Supporting information




**VIDEO S1** Upper gastrointestinal endoscopic findings on postoperative day 6. The post‐ESD esophageal ulcer demonstrated no evidence of perforation, whereas a perforation was identified in the duodenal bulb. The ulcer base appeared rigid, and endoscopic closure was deemed unfeasible.
Supporting Information
